# Long-term care: what Thailand needs?

**DOI:** 10.1186/1471-2458-14-S1-P6

**Published:** 2014-01-29

**Authors:** Nalinee Chuakhamfoo, Supasit Pannarunothai

**Affiliations:** 1 Faculty of Medicine, Naresuan University, Phitsanulok 65000, Thailand

## Background

The objective of this study was to review the literature on the definition of long-term care and systems of care that affect the quality of life of clients, operation of care providers, and the policy management in Thailand and in selected countries in Asia and Europe.

## Materials and methods

Search strategy covered online journal databases: EBSCO, PsychInfo, Routledge, BioMed Central, Blackwell, Elsevier Science, and Palliative Medicine database from 1996 to 2012, using terms related to ‘long-term care’. The selected articles included quantitative and qualitative studies related to long-term care system policies and benefits.

## Results

The study found that long-term care was defined as the care provided by formal professional health care staffs and informal staffs to people who were unable to live their lives on their own because of functional limiting health problems such as chronic illnesses in elderly people. The systems of care that affected the quality of life of clients consisted of biological, psychological and social factors. The policy management in each country was unique. Most western countries set up public payment system for long-term care to private providers, whereas most eastern countries relied on imposing filial piety values for family care with tax relief but, with limited public providers. As an Eastern country, Thailand relied on limited public providers with imposed filial piety values and tax relief.

## Conclusions

To improve the long-term care system in Thailand, available resources in the country need to be considered as the main processing factors. The long-term care policy that is able to tackle functional health limitation problems has to be managed appropriately according to the needs and economy of the country.

